# Differential roles of childhood adversities and stressful war experiences in the development of mental health symptoms in post-war adolescents in northern Uganda

**DOI:** 10.1186/s12888-014-0260-5

**Published:** 2014-09-09

**Authors:** James Okello, Maarten De Schryver, Seggane Musisi, Eric Broekaert, Ilse Derluyn

**Affiliations:** Department of Psychiatry, Makerere University College of Health Sciences, P.O Box 1962, Kampala, Uganda; Department of Psychiatry, Gulu University, P.O Box 166, Gulu, Uganda; Department of Orthopedagogics, Ghent University, H. Dunantlaan 2, 9000 Ghent, Belgium; Department of Experimental Clinical and Health Psychology, Ghent University, H. Dunantlaan 2, 9000 Ghent, Belgium; Department of Social Welfare Studies & Centre for Children in Vulnerable Situations, Ghent University, H. Dunantlaan 2, 9000 Ghent, Belgium

**Keywords:** Posttraumatic stress disorder, Depression, Anxiety, Stressful war experiences, Adolescents, Childhood adversity

## Abstract

**Background:**

Previous studies have shown a relationship between stressful war experiences and mental health symptoms in children and adolescents. To date, no comprehensive studies on the role of childhood adversities have been conducted with war-exposed adolescents living in post-war, low-resource settings in Sub-Saharan Africa.

**Methods:**

A cross-sectional study of 551 school-going adolescents aged 13-21 years old was undertaken four years post-war in northern Uganda. Participants completed self-administered questionnaires assessing demographics, stressful war experiences, childhood adversities, posttraumatic stress disorder (PTSD), depression, and anxiety symptoms.

**Results:**

Our analyses revealed a main effect of gender on all mental health outcomes except avoidance symptoms, with girls reporting higher scores than boys. Stressful war experiences were associated with all mental health symptoms, after adjusting for potential confounders. Childhood adversity was independently associated with depression symptoms but not PTSD, anxiety, and PTSD cluster symptoms. However, in situations of high childhood adversity, our analyses showed that stressful war experiences were less associated with vulnerability to avoidance symptoms than in situations of low childhood adversity.

**Conclusions:**

Both stressful war experiences and childhood adversities are risk factors for mental health symptoms among war-affected adolescents. Adolescents with histories of high childhood adversities may be less likely to develop avoidance symptoms in situations of high stressful war experiences. Further exploration of the differential roles of childhood adversities and stressful war experiences is needed.

## Background

Adolescents in Africa’s Great Lakes region continue to emerge from various armed conflicts that expose them to stressful war experiences and the risk of developing mental health symptoms e.g. PTSD, depression and anxiety [1-3]. Adolescence is a period of particular vulnerability to depression, anxiety and PTSD symptoms and many potentially stressful challenges. Studies show that direct war exposure, such as in former child soldiers, is associated with more mental health symptoms than indirect exposure to war-related events [2,4]. In addition, prolonged wars have been known to disrupt families and social support networks and thus increase childhood adversity [1,5].

More recently, some studies have explored the influence of childhood adversity and stressful war exposure on the mental health of children and adolescents following long-standing conflicts, such as in Uganda, Sri Lanka and Afghanistan [5-11]. However, there is limited data regarding the differential effects of childhood adversities and stressful war experiences on adolescents’ post-war mental health [6,12]. The impact of particular childhood adversities, such as intra-familial violence and chronic poverty, both considered risk factors for mental health in low-income countries, have not been adequately explored in Sub-Saharan Africa [1,10].

The direct correlation between the degree of trauma and the amount of mental health symptoms is consistent across a number of studies. Developmental theorists have postulated a stable association between childhood adversity and behavioral difficulties in later life [13], and some studies have provided evidence for these theories [10]. However, there is a paucity of data on whether childhood adversity adds independently to the risk associated with stressful war experiences, or if childhood adversity and stressful war experiences interact in predicting mental health.

Childhood adversity and stressful war experiences may have independent effects on mental health symptoms [8], while cumulative trauma studies show that earlier intra-familial trauma exposure is associated with worse consequences when exposed to subsequent trauma [14-17]. Whereas most studies show that stressful war experiences have an established link with post-war mental health outcomes, some studies suggest that childhood adversity might be a more potent predictor of mental health symptoms. For example, in Cabrera and others [18]’ study of post-deployment veterans, childhood adversity was a more significant predictor of depression and PTSD than combat exposure. However, some studies have found the opposite: individuals with higher exposure to developmental trauma appear less reactive to the effects of later trauma than individuals who report no such trauma [19,20]. Finally, some studies suggest that childhood adversity may moderate the effects of stressful war exposure on mental health symptoms such as PTSD and depression [18,19,21].

Childhood adversities have been proposed to modify later stress sensitivity and risk of mental health symptoms through stress sensitization (SS), stress amplification (SA), and stress inoculation (SI) models, wherein the interaction between childhood adversity and later stressful experiences determines risk for psychopathology [22]. The SS and SA models both predict that childhood adversities increase vulnerability to the negative effects of stressors occurring later in development, but they differ in the assumed expression of this vulnerability. The SS model states that childhood adversities lower the threshold for depressive reactions to recent stressors, and hence that individual differences in depression risk caused by childhood adversities are expressed in low current stress conditions in particular. Conversely, the SA model predicts that individual differences in depression risk will be expressed in high rather than low current stress conditions. In contrast to SS and SA models, the SI model posits that childhood adversities may protect against the effects of later life stress [23], because of “steeling” effects [24]. There is evidence of differences in effects of childhood adversities by gender, with SS effects seen in prepubertal boys and pubertal girls, and SA effects in prepubertal girls [22].

Taken together, these findings show that there is no single overarching principle of how childhood adversities affect mental health symptoms following stressful events later in life. In this paper, first, we aimed to assess whether childhood adversity and stressful war experiences were independently associated with PTSD, depression and anxiety symptoms. Secondly, we explore the moderating role of childhood adversity in the association between stressful war experiences and mental health symptoms. We hypothesise that (1) both childhood adversity and stressful war experiences would be associated with all mental health symptoms, and (2) childhood adversity would moderate the association between stressful war experiences and mental health symptoms.

## Methods

### Participants and procedure

As previously reported elsewhere [25,26], the study was conducted in Gulu district, northern Uganda, which until mid-2006 remained the epicentre of over 20 years of armed conflict between the Ugandan government and the rebel group the Lord’s Resistance Army (LRA). Data are derived from self-report questionnaires completed by 551 school-going adolescents in August-September 2010. Out of the 14 secondary schools in Gulu district, we purposively selected and contacted seven government and private-operated schools, with additional stratification by single (2 government, 2 private)-/mixed (2 government, 1 private)-sex schools. The study, its recruitment and consent/assent procedures were approved by the institutional review boards at Ghent and Gulu University respectively, and the Uganda national council of science and technology. Six hundred questionnaires were distributed post-consent. Overall, 24 adolescents declined or withdrew participation and 25 questionnaire sets with significant portions of missing responses were excluded, resulting in a response rate of 92% (551/600). Researchers remained present during the completion of the questionnaires, to address participants’ questions and to make sure respondents understood all items.

### Measures

Self-report questionnaires were reviewed with local mental health staff and teachers and later pretested with non-participating adolescents to ensure local validity. All questionnaires were administered in English, the official language used in all Ugandan schools. A socio-demographic questionnaire was used to determine participants’ age, gender, ethnicity, living situation, marital status of parents and whether the adolescent’s parents were still alive or not.

Childhood adversity was measured using a modified version of the Adverse Childhood Exposure (ACE) questionnaire used by Bruffaert and colleagues [27]. Six context-specific intra-familial childhood adversities: not growing up with both parents, financial adversity (i.e. dependence on food aid for at least six months), physical abuse by parents and/or other adults, sexual abuse, and witnessing fights between the parents before the age of 13 years were assessed. Although parental psychopathology, substance use and criminality are also common elements in adverse childhood experiences, we did not include them as the local experts review team advised they would be too intrusive.

Stressful war experiences were measured using a Stressful War Events (SWE) questionnaire designed specifically for the study population [28]. The SWE consists of 17 *yes/no* questions (including a final question inquiring ‘Any other stressful war event experienced?’), each referring to a stressful war event typically encountered during active conflict in northern Uganda, such as “Did you experience death of close family or friends?” Because of our interest in the experience of exposure to war events prior to 2006, when the conflict in Northern Uganda was still intense, participants were asked to limit their responses to that specific period. Childhood adversities and stressful war experiences were modelled as continuous scores in our analyses.

PTSD symptoms were assessed with the Impact of Event Scale–Revised (IES-R) [29], a 22-item scale consisting of three subscales of intrusion (8 items), avoidance (8 items) and hyperarousal (6 items). The IES-R has been administered in a number of war-affected adolescent populations in Africa, and has demonstrated good reliability and validity [4,30-32]. Respondents were asked to identify a specific event on either the ACE or SWE questionnaire as a reference point for completing the IES-R, and indicate how much it distressed them in the past month by rating each item on a 5-point scale from 0 (*not at all*) to 4 (*extremely*). Cronbach’s alpha values demonstrated good reliability (intrusion: .88, avoidance: .83, hyperarousal: .82, total IES-R score: .93).

Depression and anxiety symptoms were assessed using the Hopkins Symptom Checklist-37 for Adolescents (HSCL-37A) [33], a self-report questionnaire which had been previously used in war-affected and refugee populations [32,34]. Thirty-seven items, using a 4-point scale from 1 (*never*) to 4 (*always*), inquire about the severity of Diagnostic and Statistical Manual of Mental Disorders, Fourth Edition (DSM-IV) based symptoms. In this study, we use the subscales measuring symptoms of depression (15 items) and anxiety (10 items), both subscales showing good Cronbach’s alphas (respectively .83 and .78). For both IES-R and HSCL-37A, we used continuous scores for analyses to retain greater variability in the data.

### Data analysis

Five separate hierarchical linear regression analyses for each mental health outcome were conducted. All necessary quality criteria for regression analyses were met. In the first step, demographic variables (age, gender, mother and father still alive or not, and parent marital status) were entered. In the second step, adverse childhood experiences (ACE) and stressful war events (SWE) were added, with both covariates mean centred. In the third step, the interaction term SWE x ACE was included, followed by simple slope analysis to further explore this relationship.

## Results

### Childhood adversities, stressful war experiences and mental health symptoms

Detailed baseline characteristics of the study population and gender differences in childhood adversities, stressful war experiences and mental health symptoms are presented in Table 1. Tables 2 and 3 shows the nature and frequency of childhood adversities and stressful war experiences respectively. More girls than boys reported experiencing sexual violence and witnessing inter-parental violence and war-related experiences of lack food and water, getting wounded or disabled and not feeling accepted by family or community, although differences were relatively small.

**Table 1:** **Socio-demographic characteristics, war-related exposure, and mental health symptoms among school-going adolescents by gender**

	**Total group (N = 551)**		**Girls (** ***n*** **= 267)**		**Boys (** ***n*** **= 284)**	
	**N**	**%**	**n**	**%**	**n**	**%**
Age^‡^	16.72	1.33	16.41	1.25	17.00	1.35
**Parents alive?**						
Both parents alive	284	51.5	131	49.0	153	53.9
One parent dead	182	33.0	87	32.6	95	33.5
Both parents dead	85	15.5	49	18.5	36	12.6
**Parental marital status**						
Married	342	62.1	159	59.6	183	64.4
Divorced/separated	91	16.5	55	20.6	36	12.7
Single	118	21.4	53	19.8	65	22.9
Adverse Childhood Events (ACE)^‡^	2.49	1.45	2.67	1.52	2.33	1.37
Stressful War Events (SWE)^‡^	6.59	3.65	6.84	3.85	6.37	3.46
Depression total scores (HSCL-37A)^‡^	28.56	7.22	30.49	7.34	26.76	6.63
Anxiety symptom total scores (HSCL-37A)^‡^	19.42	5.03	20.68	5.00	18.24	4.77
PTSD symptoms total scores (IES-R)^‡^	31.22	19.5	33.84	20.2	28.77	18.50
Intrusion subscale^‡^	10.91	8.54	11.04	8.42	10.78	8.66
Avoidance subscale^‡^	11.72	8.00	11.35	7.46	12.09	8.51
Hyperarousal subscale^‡^	8.18	6.27	8.00	6.07	8.36	6.47

^‡^Mean, SD; PTSD = post-traumatic stress disorder; IES-R = Impact of Events Scale-Revised; HSCL-37A = Hopkins Symptom Checklist-37 for Adolescents.

**Table 2:** **Nature and frequency of childhood adversities by gender in war-affected adolescents**

	**Total group (** ***N*** **= 551)**		**Boys (** ***n*** **= 284)**		**Girls (** ***n*** **= 267)**		**χ** ^**2**^
	***n***	***%***	***n***	***%***	***n***	***%***	
Did not grow up with both parents	291	53.4	139	49.5	152	57.6	3.28
Experienced financial adversity	292	53.8	153	54.6	139	52.9	0.11
Experienced physical violence from parents	212	39.4	102	37.1	110	41.8	1.07
Experienced physical violence from others	286	52.8	137	49.1	149	56.7	2.80
Experienced sexual violence	41	7.5	13	4.7	28	10.5	5.92*
Witnessed inter-parental violence	253	47.2	118	42.6	135	52.1	4.50*

*p < .05.

**Table 3:** **Nature and frequency of stressful war events by gender in war-affected adolescents**

	**Total group (** ***N*** **= 551)**		**Boys (** ***n =*** **284)**		**Girls (** ***n*** **= 267)**		**χ** ^**2**^
	***n***	***%***	***n***	***%***	***n***	***%***	
Death of close family or friends	461	85.1	231	83.7	230	86.5	0.61
Forceful separation from family	170	31.7	80	29.2	90	34.4	1.41
Lived in an IDP camp	247	46.3	135	49.6	112	42.7	2.27
Witnessed violence against others	201	37.7	95	34.8	106	40.8	1.77
Victim of violence	74	13.8	32	11.7	42	15.8	1.60
Committed violence	63	11.7	28	10.2	35	13.4	1.02
Lacked food and water	310	57.9	143	52.0	167	64.0	7.40**
Lacked education	290	54.3	145	53.3	145	55.3	0.15
Lacked medical care	310	57.9	152	55.7	158	60.3	0.99
Getting wounded or disabled	160	30.8	70	26.2	90	35.6	4.91*
Lack of job or income for parents	417	77.9	215	78.5	202	77.4	0.04
Lived on streets for several months	69	13.3	33	12.1	36	14.6	0.50
Threatening by LRA	282	52.5	151	54.9	131	50.0	1.11
Not feeling accepted by family/community	212	39.6	96	35.0	116	44.3	4.40*
Taking responsibility of others/many children	230	43.6	118	43.4	112	43.9	0.00
Ever been abducted	47	9.3	21	8.2	26	10.4	0.47

*Note.* IDP = internally displaced persons; LRA = Lord’s Resistance Army.

*p < .05, **p < .01.

### Differential roles of childhood adversity and stressful war experiences

**Figure 1:**
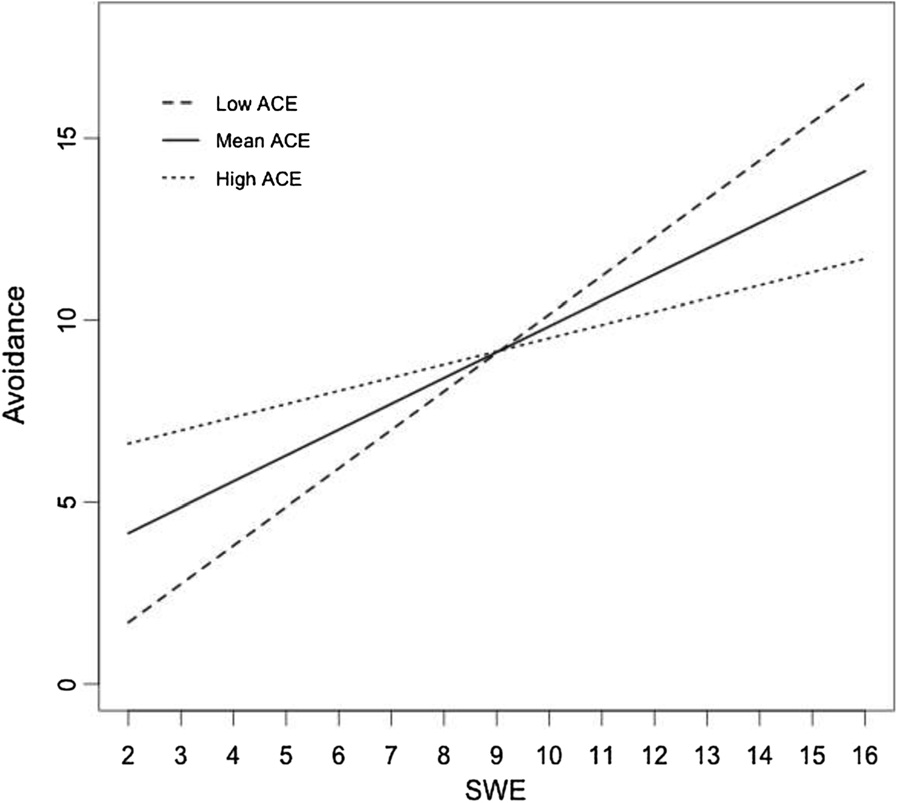
**Effect of stressful war events on avoidance symptoms, moderated by level of childhood adversities. ** Note: The regression lines represent the predicted scores, based on the final linear model for avoidance symptoms. All regression coefficients were set at 0, except for SWE and ACE. Since we assume that the relation between SWE and Avoidance is moderated by ACE, we illustrate this effect by predicting scores for 1) low ACE-scores (defined as the mean ACE-score minus 1.5 SD), 2) mean ACE-scores, and 3) high ACE-scores (defined as the mean ACE-score plus 1.5 SD). SWE= Stressful War Events; ACE= Adverse Childhood Experiences.

Hierarchical regression models revealed a main effect of gender on all mental health outcomes except avoidance symptoms, with girls reporting higher scores than boys (Table 4). Analyses showed that stressful war experiences predicted all symptoms, whereas childhood adversity only predicted depression symptoms. Lastly, a statistically significant interaction between childhood adversity and stressful war experiences was found only for avoidance symptoms. Simple slopes for the association between avoidance and war-related traumatic experience were tested for low (-1.5 SD below the mean) and high (+1.5 SD above the mean) levels of childhood adversity (Figure 1). Each of the simple slope tests revealed a significant positive association between avoidance symptoms and stressful war experiences. However, stressful war experiences were more strongly related to avoidance symptoms at low levels of childhood adversity (b = .95, SE = .14, t(438) = 6.66, p < .001) than at high levels of childhood adversity (b = .47, SE = .14, t(438) = 3.30, p < .01).

**Table 4:** **Hierarchical linear regression models: the association between stressful war experiences and mental health symptoms adjusted for sex, age, orphan status and adverse childhood events**

	**IES-R**								**HSCL-37A**			
	**Total PTSD-score**		**Intrusion**		**Avoidance**		**Hyperarousal**		**Anxiety**		**Depression**	
	***B***	***SE B***	***B***	***SE B***	***B***	***SE B***	***B***	***SE B***	***B***	***SE B***	***B***	***SE B***
Intercept	18.72	11.78	9.27	4.59	7.39	4.81	2.96	3.71	21.91***	3.13	28.60***	4.60
Age	0.81	0.66	0.16	0.26	0.32	0.27	0.28	0.21	-0.09	0.18	-0.03	0.26
Sex *(base = M*)	4.06*	1.75	1.78**	0.69	0.47	0.73	2.06***	0.55	2.32***	0.47	3.63***	0.69
Mother alive? *(base = no)*	-3.91	2.37	-2.80**	0.93	-0.52	0.98	-0.63	0.74	-1.80**	0.63	-1.43	0.92
Father alive? *(base = no)*	-0.80	1.88	-0.53	0.74	0.16	0.78	-0.20	0.59	-0.67	0.51	0.41	0.75
Parent marital status *(base = divorced or separated)*												
- *Married*	0.76	2.27	0.33	0.88	-0.42	0.94	0.49	0.71	-0.18	0.61	-0.87	0.90
- *Single*	-0.38	2.88	0.18	1.13	-0.88	1.20	-0.13	0.91		0.77	-0.66	1.14
ACE	0.78	0.70	0.18	0.27	0.40	0.29	0.28	0.22	0.27	0.19	0.84**	0.27
SWE	2.39***	0.26	0.96***	0.10	0.71***	0.11	0.65***	0.08	0.23***	0.07	0.35***	0.10
ACE x SWE	-0.14	0.15	0.03	0.06	-0.17**	0.06	-0.01	0.05	0.06	0.04	0.08	0.06
ΔR^2^ step 1	.09***		.11***		.04***		.09***		.13***		.12***	
ΔR^2^ step 2	.19***		.19***		.12***		.15***		.04***		.07***	
ΔR^2^ step 3	.01		.00		.01***		.00		.01		.01	
R^2^	.29***		.30***		.17***		.24***		.18***		.20***	

*Note*. Results of hierarchical linear regression analyses predicting mental health symptoms: In the first step, demographic variables (age, gender, mother and father still alive or not, and parent marital status) were entered; in the second step, ACE and SWE were added; in the third step, the interaction term SWE × ACE was included. Regression coefficients *(B and SE B)* from the last step in the analyses are shown.

ACE = adverse childhood experiences; PTSD = post-traumatic stress disorder; IES-R = Impact of Events Scale-Revised; HSCL-37A = Hopkins Symptom Checklist-37 for Adolescents.

*p < .05, **p < .01, ***p < .001*.*

## Discussion

The present study aimed to examine the differential effects of childhood adversities and stressful war experiences on mental health symptoms and to explore the moderating role of childhood adversity in the relationship between stressful war experiences and mental health symptoms. There are three notable findings in this study. First, there was a main effect of gender on all mental health symptoms except avoidance symptoms, with females reporting higher scores than males. Females also reported higher scores of childhood adversities, stressful war experiences and PTSD, depression and anxiety symptoms than males. Although the findings may reflect a gender-specific exposure and response to particular traumas, suggesting that war is more harmful to females than males, the higher rates of exposure of females to both war-related and intra-familial experiences could have led to an elevation of symptom response [35].

Second, in keeping with our hypothesis and the extant literature, stressful war experiences were risk factors for all mental health symptoms [1-4]. Contrary to the hypothesis, childhood adversities were associated with risk of developing only depression symptoms. Although the extant literature has linked adverse childhood experiences to PTSD [6-9], some studies show that childhood adversities are associated with vulnerability to depression symptoms in post-conflict settings [18]. In contrast, Entholt and Yule [36] found that PTSD symptoms were more strongly linked to earlier war-related trauma, whereas depression symptoms were more strongly linked to recent stressful life events among post-war youth. While we acknowledge that not all childhood adversity and stressful war experiences items qualify as trauma in the DSM-IV, and that childhood adversity may be qualitatively different from war-related trauma in general, the results suggest that depression may develop similarly across different forms of exposure, from wars to childhood adversities [37].

Childhood adversities were not associated with PTSD and anxiety symptoms in our study sample. This finding is inconsistent with that of other researchers who have found stressful war experiences to be less predictive of mental health symptoms than childhood adversities [18]. Although emerging data suggests that non-traumatic and adversity events may be associated with PTSD [38], further research is needed to delineate the specific effects of childhood adversity on PTSD and other mental health outcomes among post-war adolescents.

Lastly, in contrast to our hypothesis, childhood adversities did not moderate the association between stressful war experiences and all mental health symptoms, but it moderated the association between stressful war experiences and avoidance symptoms. Specifically, stressful war experiences were significantly less predictive of avoidance symptoms in situations of high levels of childhood adversity compared to situations of low levels of childhood adversity. This finding contradicts previous studies in which high childhood adversity was reported to increase vulnerability to the effects of stressful war experiences [14-17]. Two previous studies have found similar interaction between childhood adversity and stressful war exposure, in which individuals with higher exposure to childhood adversities appeared less reactive to the effects of stressful war exposure than individuals who reported no such prior exposure [18,19]. Plausible explanations for this finding have been suggested [18,19,21]. One hypothesis is that adolescents exposed to childhood adversities display lower reactivity to stressful war experiences [18,19]. Second, previous trauma may act to create a symptom “ceiling effect”, in which only the most distressing symptoms are reported following stressful war experiences, although they may be manifesting more symptoms than they are likely to display [18]. In keeping with established theory, this finding lends support for the stress inoculation model in which a history of childhood adversity protects against the effects of later stress [22,23].

We draw particular attention to the fact that childhood adversity did not moderate the relationship between stressful war experiences and the common mental health symptoms among adolescents post-war. Regardless of the level of childhood adversities, stressful war experiences remained predictive of depression, anxiety and PTSD symptoms, in keeping with the extant literature [1-3]. Possible explanations for this could be that stressful war experiences retain their potency irrespective of the severity of childhood adversity, whose effects may wane over time. It remains unclear why childhood adversities would significantly moderate the association between stressful war experiences and avoidance symptoms but not other PTSD cluster symptoms.

### Limitations

The data were retrospective and self-reported, and consequently subject to recall and social desirability biases. Although recall bias is expected to be less extreme in a survey of adolescents than adults or younger children, recall bias is still a possibility in retrospective reports of childhood adversities and stressful war experiences. The cross-sectional design precludes both causal inference (as event reporting may be confounded by current psychological functioning and age) and the longitudinal analysis of adjustment trajectories [38]. The generalizability of our findings is limited to only school-going adolescents. Furthermore, given that active war ended in 2006, the extended time frame (four years post-war) created a situation in which post-war factors, such as post-war trauma could occur and compete for explained variance. The time frame also sampled youths 9 to 17 years old at the end of the war – a group that was possibly less severely exposed than older adolescents. This may have led to low endorsement rates for some event types (e.g., physical violence) that prevented their predictive effects from being adequately tested [39]. As a consequence, factors such as daily stressors and more recent traumatizing events, or parental and peer attachment, might have influenced mental health symptoms [37]. Fifth, our coverage of childhood adversities was not exhaustive. For purposes of this study, and to avoid participant fatigue, we focused specifically on family-related adversities, consistent with previous work [27], but numerous other adversities are associated with elevated risk for mental health symptoms. Non-violent [40] and stressful war events such deprivation of food and water and being forced to perform rituals [2] have been linked to PTSD, but they may not constitute DSM-IV qualifying trauma. Further, the psychometric properties of the Stressful War Events Questionnaire are unknown. The measure was developed with the assistance of local expert teams familiar with the types of war events children experienced in Uganda. However, it is possible that some important traumatic events (e.g., witnessing (re)burial of loved ones) were not listed on the questionnaire. Lastly, using count scores summing up conceptually diverse childhood adversities and stressful war experiences assumes an arithmetic relationship between these experiences, an assumption which may not be valid. Although this is in accordance with earlier studies [41] stating that both low- and high-threat events increased the risk for mental health symptoms, and that counts of events (without regard to type of trauma) predicted outcomes, no consensus exists yet on methods of weighting aspects of traumatic exposure [39].

## Conclusions

The above limitations notwithstanding, the data and analyses reported here add further to the literature on post-war adolescents by replicating and extending previous research on the mental health symptoms and the impact of exposure to both childhood adversity and stressful war experiences. Assessing and understanding the role of moderating influences of childhood adversity is important for identifying individual differences in the influence of stressful war experiences on mental health, and for targeting interventions that will hopefully improve mental health outcomes in conflict and post-conflicts situations. Our findings thus support the differential associations of childhood adversities and stressful war experiences with post-war mental health symptoms [11,42]. Although the impact of childhood adversity has received recent attention in post-conflict settings, the result of this study draws attention to the impact of stressful war experiences and the need to adopt a developmental trauma perspective [43]. Further research is needed to develop more clearly theoretical models concerning the relationships among childhood adversities, stressful war experiences and mental health outcomes. In particular, there is a need to better understand the mechanism(s) through which childhood adversities may serve as both vulnerability and protective factors for and against the negative effects of stressful war experiences.

## Abbreviations

PTSD: Post traumatic stress disorder
SS: Stress sensitization
SA: Stress amplification
SI: Stress inoculation
IDP: Internally displaced persons
LRA: Lord’s resistance army
ACE: Adverse childhood exposure
SWE: Stressful war events
IES-R: Impact of events scale-revised
HSCL-37A: Hopkins symptom checklist-37 for adolescents
DSM-IV: Diagnostic and statistical manual of mental disorders, fourth edition
